# Predictors of teen sexual behavior

**DOI:** 10.1016/j.childyouth.2023.107247

**Published:** 2023-10-21

**Authors:** Andrew Langan, Marina Mileo Gorzig

**Affiliations:** Mathematica, 600 Alexander Park, Suite 100, Princeton, NJ 08540, USA

**Keywords:** Sexual behaviors, Sexual initiation, Sexual health

## Abstract

**Purpose:**

To identify characteristics and behaviors among teenagers that predict sexual initiation or sexual activity and to evaluate alternative methods for predicting sexual behavior among teenagers.

**Methods:**

We used longitudinal data from an evaluation of the Making Proud Choices! teen pregnancy prevention program. The evaluation, funded by the Office of Population Affairs, assessed academic years 2016–2017 through 2018–2019 and examined 2,138 Grade 9 and 10 students of all genders. We used ordinary least squares (OLS), least absolute shrinkage and selection operator (lasso), and stratified OLS to identify behaviors and characteristics at baseline that predict sexual initiation, recent sex, and sex without a condom.

**Results:**

OLS and lasso regression show that pre-sexual behaviors and substance use are the most powerful predictors of sexual initiation among teens. Lasso additionally identified higher-order interactions between predictors of sexual activity, including variation in predictors’ influence across population subgroups. Stratified OLS regression predicted behavior most accurately for sexual initiation, recent sex, and sex without a condom. However, stratified OLS also reduces the sample size, and therefore precision, for each regression.

**Conclusions:**

Current behavior, not knowledge or beliefs about sex, best predicts future behavior. It can be difficult to evaluate the impact of programs designed to delay sexual initiation among younger adolescents because sexual behaviors often occur at older ages. Our results suggest that pre-sexual behaviors are strongly predictive of subsequent sexual initiation and having sex without a condom, so these could be used as outcome measures for identifying high-risk students or evaluating interventions among younger adolescents. Additionally, our results show that lasso can be a useful technique to identify subgroup differences in the relationship between predictors and future sexual activity and for prioritizing variables to collect in a survey.

## 1. Introduction

Sexual behavior and exploration are common and normal during adolescence (O’Sullivan and Thompson, 2014), but early sexual initiation is related to negative outcomes for youth (Centers for Disease Control and Prevention, 2021). Starting to have sex at a younger age is associated with increased risk of sexually transmitted infections (STIs) (Coker et al., 1994), unintended pregnancies, and perpetrating sexual violence (O’Donnell et al., 2001). Unintended pregnancy among teens is associated with decreased educational attainment for teens and their children (Perper et al., 2010; Maynard, 1996). Among high school-aged youth who participated in the 2019 Youth Risk Behavior Surveillance System, 38 % report having had sex (19 % of Grade 9 students and 57 % of Grade 12 students) (Centers for Disease Control and Prevention, n.d.). Although sexual activity rates among teenagers have fallen in the last two decades (Ethier et al., 2018), racial and ethnic disparities persist in access to and use of high-quality birth control (Szucs et al., 2020). Reflecting this disparity, teen birth rates among Black, Hispanic, American Indian, and Alaska Native teens are more than double those of White teens (Tollestrup, 2022). Identifying the characteristics and behaviors of youth more likely to have sex at a young age or without birth control could help educators engage these youth *before* they initiate this activity.

More broadly, identifying predictive characteristics and behaviors could help researchers evaluate sex education interventions that focus on younger students or design effectiveness studies that offer a short time frame in which outcomes can transpire (Coyle and Glassman, 2016). For instance, the research clearinghouse for the U.S. Department of Health and Human Services’ Teen Pregnancy Prevention Evidence Review focuses on sexual behavior, STIs, and pregnancy (Mathematica, n.d.). Identifying precursors to early sexual initiation or higher-risk sexual activity could broaden these standards and the range of evaluable interventions.

Teenagers who engage in early or higher-risk sexual behavior often differ greatly from those who do not on a variety of characteristics and behaviors. For example, Coyle et al. (2014) used detailed data on pre-sexual behaviors and found that touching a partner’s genitals, having oral sex, being in a relationship, and going on dates alone are all correlated with having vaginal sex. Likewise, higher-risk sexual activity is associated with poor academic achievement, lower engagement in school (Halpern et al., 2000; Resnick et al., 1997), and sexting (Mori et al., 2019).

Importantly, these findings are from cross-sectional analyses that identify contemporaneous relationships. Predicting future sexual behavior among teens requires data on the same teenagers over time (Buhi and Goodson, 2007). Cross-sectional comparisons are impacted more strongly by confounding variables or simply identify other factors contemporaneous with sexual activity, making it harder to identify factors that actually predict sexual behavior.

Longitudinal analyses better predict behaviors than cross-sectional analyses. However, longitudinal analyses of teen sexual activity often focus on how specific baseline behaviors and characteristics relate to later sexual activity. For example, online sexual activity and engaging with more sexual media online predict HIV infection, intimate partner violence, sexual assault (Maas et al., 2019), and earlier sexual initiation (Brown et al., 2006). Similarly, religiosity during adolescence is associated with lower rates of sexual activity in later adolescence and early adulthood (Vasilenko and Espinosa-Hernández, 2019), and attitudes about sex (Carvajal et al., 1999; Blinn-Pike et al., 2004; Goesling and Rangarajan, 2008; Trenholm et al., 2007) predict future sexual behavior, such as initiating sex or having sex without a condom. There is a lack of longitudinal analyses that analyze how a broad range of precursor behavior is related to later sexual activity.

Additionally, improved access to data and lower computational costs have made machine learning techniques accessible to more researchers. Machine learning methods are useful for identifying complex or unexpected relationships between predictors and outcomes, including analyzing the relationship between baseline characteristics and future behavior. The least absolute shrinkage and selection operator (lasso) method identifies the most predictive explanatory variables from a regression with many controls (Tibshirani, 1996). Lasso has been used to predict risky behavior among adolescents including pregnancy and arrest (Ando et al., 2022), problematic social media (Reem et al., 2021), and self-injury (Smith et al., 2020; Uh et al., 2021). Among adults, lasso has been used to predict sexual behavior (Sherafat-Kazemzadeh et al., 2021) and hepatitis B infection (Guo et al., 2015). Lasso enables researchers to identify individual variables and combinations of variables that are effective predictors of behavior, which can help identify moderating or amplifying interaction effects or subgroup differences in the influence of covariates (Zhao et al., 2022). Comparing how well lasso predicts behavior compared to traditional techniques can help researchers evaluate the benefit of increased accuracy relative to the cost of increased computational demand and reduced intuitive interpretation of the results.

### Study Aim 1:

We used detailed, longitudinal data to examine what factors predict subsequent sexual initiation and sex without a condom using baseline pre-sexual or sexual behaviors and other factors.

### Study Aim 2:

We assessed the usefulness of lasso, a machine learning technique, to predict sexual activity among our sample.

### Study Aim 3:

We examined whether lasso’s accuracy varies by subgroup and how much accuracy improves within subgroups when using traditional ordinary least squares (OLS), lasso, and stratified OLS regressions.

## 2. Methods

### 2.1. Participants

We used data from the experimental impact evaluation of the Making Proud Choices! (MPC) teen pregnancy prevention program, which was funded by the Office of Population Affairs and received approval from the WCG New England Institutional Review Board. The impact of MPC on sexual activity was evaluated in Cole et al. (2022) so we do not discuss the details of the program itself in this paper. The evaluation ran from academic years 2016–2017 through 2018–2019, and included 31 cohort clusters of students from 15 high schools in four cities with above-average rates of teen births and STIs.

The total MPC evaluation sample included 2,138 students. We analyze the 1,304 students from that sample with complete data on at least one outcome variable and all relevant covariates. These students are almost all from Grade 9 or 10, and span both the experimental treatment and control groups.^1^ Among our sample students, 78 % identified their race as Black, and 30 % reported prior sexual experience at the time they entered the study. The sample was 41 % boys, 57 % girls, and 1 % who identified as transgender or “other.”^2^ The data and instruments are available by request from the Office of Population Affairs.

### 2.2. Data collection

At baseline, students reported pre-sexual or sexual behaviors, attitudes and beliefs about sex, risk-tasking behavior, and demographic information. For students who were sexually inexperienced at baseline, the survey used skip logic to obtain detailed information about pre-sexual behaviors, rather than asking a series of questions about sexual behaviors they had not engaged in. After the MPC curriculum was delivered, students completed the follow-up survey, an average of 8.6 months after the baseline data collection. The follow-up survey contained the three outcome variables of interest: sexual initiation, recent sex (within the past 30 days), and recent sex without a condom.

### 2.3. Data analysis

We identified predictors of sexual behavior using two statistical methods: we use OLS multivariable regression to address Aim 1 and lasso to address Aim 2; for Aim 3, we compare the accuracy of the two approaches.

Approach for Aim 1: We first used a traditional OLS multivariable regression to show which baseline variables predict sexual behavior at follow-up. We regressed each outcome variable (sexual initiation, recent sex, and recent sex without a condom measured at follow-up) on the baseline covariates shown in Table 1 along with measures of parental presence and education, additional risky non-sexual behaviors, past sexual health outcomes, and questions about sexual health knowledge, attitudes, and beliefs plus cluster fixed effects.^3^ Online Appendix Table A.1 we define selected pre-sexual and sexual behaviors; Cole et al. (2022) defines all covariates and other outcomes in detail. Our sample for initiation included only students who were sexually inexperienced at baseline. We limited our sample to those students who provided valid responses for each covariate and at least one outcome of interest. We used this regression to predict the probability that an individual student would engage in the three outcomes by the time of the follow-up survey. By comparing the prediction with their actual behavior, we calculated the proportion of students whose behavior we predicted correctly.

Approach for Aim 2: We then built on this approach to examine whether any precursors appear more important when considered for specific subgroups or in combination with other variables. Specifically, we used lasso to evaluate whether any of the two-way interactions between our variables gave additional predictive power by selecting between both main effects and interactions. Lasso conducts repeated regressions using a randomly selected subset of the data and selects the control variables whose coefficients have the largest standardized beta (in absolute value). It then checks the prediction error for the resulting model on the remaining data left out of the first analysis sample. It repeatedly analyzes random samples in this way to help researchers arrive at a short list of variables whose predictive power holds up well across repeated subsamples. Lasso is particularly useful when there are many variables and including all two-way interactions would reduce the degrees of freedom or statistical power of the regression. Lasso enables researchers to select main effects and interactions that provide the most predictive power. As before, we used the regression to predict the probability that an individual student would engage in the three outcomes by the time of the follow-up survey and calculated the proportion of students whose behavior we predicted correctly.

Approach for Aim 3: We stratified the OLS regression by gender, race, grade level, and sexual orientation and again used the regression to predict individual student’s behavior at the time of the follow-up survey. We then compared the proportion of student behavior that was predicted correctly by pooled OLS, lasso, and stratified OLS for each group. It is typically infeasible to stratify a regression for all subgroups. However, doing so provides an illustrative comparison for the value lasso adds. If the predictive abilities of lasso and stratified OLS values are similar, lasso provides a more efficient way to analyze subgroups. Rather than researchers selecting subgroups arbitrarily, lasso identifies which interactions matter most for prediction. We included an alternative version of the lasso model to evaluate the marginal increase in predictive ability when we used the variables lasso selected. We also included “implicit main effects,” which are variables lasso selects as part of an interaction term but where lasso did not select the variable itself.

## 3. Results

### 3.1. Study Aim 1: Identifying baseline behavior that predicts subsequent sexual activity

The results of estimating the OLS regression show that sexual and pre-sexual activity at baseline are predictive of sexual activity at follow-up. The column labeled “Initiation” in Table 2 shows that sexual initiation among sexually inexperienced students was more common among students who engaged in more pre-sexual behaviors and who were in a serious relationship. The columns labeled “Recent sex” and “Sex without a condom” show that having sex at baseline, sexual frequency, and relationship status at baseline are associated with increased probability of having recent sex and sex without a condom at follow-up.

Timing factors, such as summer break and grade level, are also important in predicting sexual initiation, recent sex, and sex without a condom. Summer between baseline and follow-up is positively associated with all three outcomes. This relationship is largest for sexual initiation among those who are sexually inexperienced. Among those sexually inexperienced, Grade 9 students are more likely to initiate sex by the time of the follow-up survey. This suggests that among those students who will have sex at some point in high school, Grade 9 is a common time to start.

### 3.2. Study Aim 2: Assessing usefulness of lasso for predicting sexual activity

Lasso chose among the baseline characteristics, cluster fixed effects, and two-way interactions to select controls for the regression. We examined 46 baseline characteristics (43 for sexual initiation); none of these main effects were selected for sexual initiation, three were selected for recent sex, and two were selected for sex without a condom. Out of the 46*46 = 2,116 potential interactions (1,849 for sexual initiation), 31 were selected to include in the regression for initiation, 12 for recent sex, and seven for sex without a condom. Table 3 shows the coefficients of the selected variables. The interactions included by lasso do not suggest a strong role for moderating or amplifying interaction effects. Rather, many appear to be arbitrary combinations of important variables that efficiently increase the predictive power of the model. Some variables appear many times—for example, the substance use scale appears in many interactions across all three outcomes. These results show some predictive role for factors such as knowledge, beliefs, and attitudes, but only through interactions with behaviors.

In addition, the selected interactions show differences in the relationship between baseline variables and sexual behaviors by subgroup. For example, in both the OLS regression and the lasso analysis, a student’s level of substance use was positively related to sexual initiation (*p* =.036 in the OLS model). However, the lasso model shows that this relationship varied with a number of other student characteristics and behaviors. For instance, one of these interactions suggests that substance use has a systematically different association with initiation for White students than students of other races, when combined with the other interaction terms selected by lasso.

### 3.3. Study Aim 3: Comparing the accuracy of predictions by different models

We used the OLS and lasso regression results to predict each individual student’s probability of initiating sex (among the sexually inexperienced), having recent sex, and having sex without a condom. To evaluate the accuracy of the prediction, we compared the predicted behavior at follow-up with actual behavior. We dichotomized the prediction—if a student had a 0.5 probability or higher for an outcome, we predicted the student would engage in the outcome activity (sexual initiation, recent sex, or sex without a condom). If a student was below the 0.5 cutoff, we predicted they would not engage in that activity at the time of follow-up.

In Table 4, we show the proportion of students whose behavior we predicted correctly, for the full sample and for separate subgroups. Overall, OLS accurately predicted sexual initiation for 81 % of students, recent sex for 81 % of students, and sex without a condom for 85 % of students. The accuracy of these predictions varied by the demographic attributes of the student. The percentage of students we predicted correctly was lower for boys and Grade 10 students for all three outcomes. Predictions were less accurate among Black students for two outcomes, initiation and recent sex.

The accuracy of the lasso-selected model declined slightly for sexual initiation and sex without a condom; although lasso selects variables to maximize the overall predictive power of the regression, using the predicted probability to model a dichotomous behavior was not more accurate than OLS. As Table 5 shows, most students’ predicted behavior remained the same between the standard multivariable model and the lasso-selected model. For initiation, 6.9 % of students’ predicted behavior changed between the two models; likewise, only 4.8 % of predicted behavior changed for recent sex and 5.4 % for sex without a condom.

The accuracy of the lasso predictions varied by students’ demographic attributes. The proportion of boys we predicted correctly for sexual initiation and sex without a condom decreased slightly with lasso, while increasing slightly for girls. The proportion of Black students whose outcomes we predicted correctly also declined slightly with the lasso-selected model for all three outcomes. Lesbian, gay, and bisexual (LGB) students are largely not impacted by model selection. However, for sex without a condom, lasso decreased the proportion predicted correctly.

We used two alternative regressions to evaluate the predictive abilities of OLS and lasso models. First, we added all the individual variables that lasso selected as interactions—for example, when lasso indicated that the substance use variable interacted with the White variable, we included the interaction and added both substance use and White variables to the regressions. Table 4 refers to this regression as “lasso with implicit main effects.” As expected, by using more variables, the predictions remain similar or improve slightly relative to the traditional lasso-selected model.

Second, we stratified the regression by gender, race, and grade level—we estimated the OLS regression separately for subgroups and used the group-specific regression to predict each student’s behavior. Stratifying regressions increased the proportion predicted correctly across all outcomes and for almost all subgroups relative to the joint OLS model. In particular, stratifying by gender increased the proportion of boys we predicted correctly across all outcomes. The proportion correct among non-Black students increased across all outcomes but remained similar to the pooled OLS for Black students. The proportion predicted correctly for Grade 10 students increased for recent sex and sex without a condom.

## 4. Discussion

Our first aim in this study was to examine what baseline behaviors and characteristics predicted subsequent sexual behavior. We found attitudes and beliefs did not strongly predict later sexual behavior. Rather, substance use and pre-sexual activity predicted sexual initiation. Likewise, baseline sexual behaviors, not knowledge about condoms, predicted sex without a condom. Because of these predictive relationships, we can use the former behaviors as an early indicator of the latter.

Having sex at a young age and experiencing unintended pregnancies are associated with negative long-term outcomes for youth (Centers for Disease Control and Prevention, 2021; Coker et al., 1994; O’Donnell et al., 2001; Perper et al., 2010; Maynard, 1996). Identifying early behaviors that predict higher-risk activity can help educators focus teen pregnancy prevention interventions on youth engaging in those precursor behaviors and provide early metrics for assessing sex education curricula among sexually inexperienced youth (Coyle and Glassman, 2016). However, we caution against targeting precursor behaviors, because many are developmentally appropriate (O’Sullivan and Thompson, 2014); targeting precursor behavior rather than sexual behaviors associated with negative later outcomes may have unintended developmental consequences. Instead, we suggest using precursor behavior to identify students who may benefit from access to safer sex materials and teen pregnancy prevention curricula.

Our analysis shows that summer break is a strong predictor of all three sexual behavior outcomes, including having sex without using a condom. Sex without condoms might be more likely during this time, when youth have less access to school resources, including safer sex materials distributed at school, and less access to friends and mentors. Schools and health care workers that engage with youth could consider whether there is sufficient access to safer sex products during summer and how to provide students with what they need, or whether more intentional outreach about sexual health should happen before summer break begins.

Our second study aim was to assess the value of using machine learning models like lasso to predict teen sexual behavior, including lasso and stratified OLS regression. When conducting predictive analyses, tools that acknowledge the heterogeneity in how predictors affect outcomes across youth with different backgrounds, attitudes, or other characteristics can improve predictive accuracy or the parsimony of the model. In our analysis, the lasso models tended to identify a small number of apparently predictive interactions, whereas main effects tended to be less important. With increased computing power and more machine learning techniques being programmed into many statistical packages, lasso is now more accessible to many researchers. The results of lasso are also interpreted in a similar way as traditional OLS regression and easier to understand and apply than some machine learning techniques.

Our third aim was to assess how well the different statistical models predicted future behavior. We found that none of the three models perfectly predict future sexual behavior. Stratified OLS regressions predicted sexual activity at follow-up most accurately, possibly because they lift the constraint that covariates exert the same influence across all groups. However, they necessarily reduce sample size and efficiency relative to a pooled OLS regression. Lasso could help identify a limited set of covariates for which an unconstrained specification is appropriate. For example, the lasso model shows that the positive relationship between substance use and sexual behavior varies with a number of other student characteristics and behaviors. Analysts using these data to predict subsequent initiation could possibly improve their predictions by including this interaction term in their specification, without sacrificing efficiency and precision by splitting the sample to analyze students separately by race.

However, there was little improvement in predictive accuracy between traditional OLS and lasso, and both performed worse than stratified OLS. Our results do not suggest that lasso should be used instead of OLS, but rather that lasso can be considered due diligence for researchers. It might identify important interactions and increase the predictive ability of the model or identify few interactions and therefore increase confidence in the main results. In addition, lasso identifies the most predictive variables, which could help researchers prioritize questions on their survey instruments. Shorter surveys increase completion rates, improve samples, and reduce participants’ time cost.

The accuracy of predictions varied somewhat among different demographic groups. We predicted behavior correctly for a slightly lower proportion of boys, Grade 10 students and, for some outcomes, Black students. The accuracy for different subgroups also varied by the model selected. This suggests that the relationship between predictors and outcomes are less clear for students in these groups; it is possible there is other information that could be included in data collection that would more accurately predict future behavior for students in these groups. Additionally, adolescents have lower accuracy of self-reported sexual behaviors than adults, and this accuracy varies by demographic subgroup (Brown et al., 2012; DiClemente et al., 2011). Measurement error in sexual activity at baseline or follow-up could impact the predictive ability of the model. Practitioners should keep these limitations in mind when they apply the results of these analyses. If results are being used for high-stakes decisions, practitioners should consider their knowledge of individual students and not fully rely on the predictive model—particularly for students who are members of groups whose behavior is predicted with more error.

Researchers should consider using lasso for analyses with many variables. Lasso makes it easy for researchers to identify which variables—including both direct effects and interaction terms—to include in their analysis to maximize predictive power. However, our results also suggest caution when using lasso to predict dichotomous behaviors. Traditional OLS had more accurate predictions than the lasso-selected models for sexual initiation and sex without a condom for the whole sample and within most subgroups. Stratified OLS regression had the most accurate predictions across sexual initiation, recent sex, and sex without a condom for almost all subgroups.

## 5. Strengths and limitations

A clear strength of this study is having detailed longitudinal data on teen demographics and behaviors. Because of these detailed data, we are able to compare different methods of using baseline behavior and characteristics to predict subsequent sexual initiation or sex without a condom. However, there are weaknesses to our approach. Our sample is not representative of all Grade 9 and 10 students in the U.S.—the locations were selected because they had above-average rates of teen births and STIs—so our results are not generalizable. Additionally, adolescents sometimes inaccurately report sexual behaviors, and accuracy of self-reported sexual behavior varies by subgroup (Brown et al., 2012; DiClemente et al., 2011). This measurement error complicates the interpretation of the outcome variables considered and the analysis of predictive power by subgroup.

Finally, if pre-sexual behaviors are to be used as outcomes for evaluating the effectiveness of an intervention involving younger adolescents, researchers should consider the intervention’s underlying mechanisms. The results in this study suggest that a decrease in pre-sexual behavior would precede a decrease in sexual initiation. However, reducing pre-sexual behavior might not be necessary for reducing sexual behavior—for instance, some interventions might work by reducing the likelihood that youth would transition from pre-sexual to sexual behavior. Additional research would the clarify the relationship between changes in precursors and downstream behaviors.

## Supplementary Material

Supplementary

## Figures and Tables

**Table 1:** Descriptive statistics showing selected baseline characteristics and outcomes.

	Mean	Std dev
*Baseline variables n* = *1,304*		
Age	15.56	0.82
Grade 9	0.44	0.50
Grade 10	0.51	0.50
Gender: Girl	0.57	0.49
Gender: Boy	0.41	0.49
Only or mostly attracted to same-gender people	0.05	0.21
Only or mostly attracted to different-gender people	0.82	0.38
Attracted to both male and female gender people equally	0.08	0.28
Other sexual orientation	0.03	0.18
Black	0.78	0.40
Latino	0.03	0.16
White	0.11	0.31
Multiracial	0.09	0.29
Other race	0.02	0.13
Ever skipped school	0.19	0.39
Substance use scale	0.10	0.20
Relationship status: Single	0.53	0.50
Relationship status: Casually dating	0.21	0.41
Relationship status: Serious relationship	0.22	0.41
Relationship status: Engaged	0.04	0.18
Ever had sex	0.30	0.46
Had sex in last three months	0.18	0.39
How many times had sex in the last three months	1.53	8.23
Number of partners (vaginal sex), last three months	0.79	3.42
Had sex without a condom, last three months	0.13	0.34
How many times had sex without a condom, last three months	0.91	7.23
Had vaginal sex without birth control, last three months	0.09	0.29
How many times had vaginal sex without birth control, last three months	0.49	4.38
Summer between baseline and follow-up survey	0.53	0.50
*Pre-sexual behaviors (n* = *833)* * Variables are on a [0,1] scale where higher scores represent more of a given activity*		
Sexting scale	0.32	0.32
Touching scale	0.24	0.37
Opportunity scale	0.48	0.38
Kissing scale	0.49	0.43
Ever been in a relationship	0.80	0.40
Pressure scale	0.40	0.36
*Outcome variables*		
Sex in the last three months (n = 1,294)	0.29	0.46
Sex without a condom in the last three months (n = 1,282)	0.20	0.40
Initiated sex (among the sexually inexperienced) (n = 833)	0.24	0.43

Notes: Observations include the analytic sample—those with complete baseline data and data for at least one outcome. Cole et al. 2022 define covariates and outcomes in detail. Pre-sexual behavior categories include aggregated responses to questions about sending or receiving nude photos, touching, opportunities for having sex (has ever hung out alone with or lain with someone they were attracted to), kissing, self-described relationship history, and feeling peer pressure to have sex.

**Table 2:** Ordinary least squares regression results for sexual initiation, recent sex, and sex without a condom: Important predictors (statistically significant or having a standardized coefficient of 0.10 or higher).

Initiation			Recent sex	Sex without a condom
**Substance use scale**	0.202**	**Ever had sex**	0.364***	0.257***
	(0.0964)		(0.0361)	(0.0332)
**Summer between baseline and follow-up**	0.131***	**Substance use scale**	0.273***	0.220***
	(0.0502)		(0.0600)	(0.0549)
**Grade 9**	0.131*	**Had sex in last three months**	0.202***	0.0606
	(0.0766)		(0.0588)	(0.0545)
**Touching scale**	0.126***	**Been pregnant or gotten someone pregnant**	0.135	0.168*
	(0.0485)		(0.110)	(0.100)
**Opportunity scale**	0.123**	**Summer between baseline and follow-up**	0.0800**	0.0783**
	(0.0478)		(0.0363)	(0.0332)
**Sexting scale**	0.120**	**In a serious relationship**	0.0625**	0.0445*
	(0.0523)		(0.0289)	(0.0263)
**Refusal skill**	0.118**	**In a casual relationship**	0.0496*	0.0380
	(0.0538)		(0.0277)	(0.0252)
**In a serious relationship**	0.107**	**How many times had sex in past three months**	0.00893**	0.0134***
	(0.0428)		(0.00380)	(0.00375)
**Girl**	−0.102***	**Grade 9**	−0.0324	−0.101**
	(0.0371)		(0.0563)	(0.0511)
		**Age**	−0.0334	−0.0380**
			(0.0210)	(0.0192)
**Other BL predictors**	X		X	X
**n**	833		1,294	1,282
**R-squared**	0.211		0.363	0.314
**Share predicted correctly**	0.806		0.811	0.854

Note: Other baseline predictors include those listed in Table 1 (omitted categorical variables include not being in a relationship, being a boy, and being attracted to different-gender people) and cluster fixed effects.

Students who have not had sex are asked questions about pre-sexual behaviors that students who have had sex are not asked. Regressions for initiation include students who have not had sex at baseline. They include pre-sexual behavior as covariates but not sexual behavior, because these students have not had sex. The regressions for recent sex or sex without a condom include all students and therefore include sexual behaviors as covariates but not pre-sexual behavior, because sexually experienced students were not asked all of these questions.

**Table 3:** Predictor variables and interaction terms selected by lasso, with coefficients and statistical significance.

Initiation		Recent sex		Sex without a condom	
Attracted to both male and female gender people × Hispanic	0.396	Ever had sex	0.297***	Ever had sex	0.172***
Attracted to both male and female gender people × Mother’s education: Some college or more	0.0562	Had sex in the past three months	0.118	Had sex in the last three months	0.0823*
Attracted to both male and female gender people × Father’s education: Some college or more	0.231	Substance use scale	0.106		
White × Substance use scale	0.519**	Attracted to same-gender people × Knowledge about other contraception	0.593***	Father figure present × How many times had sex in last three months	0.00386
Multiracial × Beliefs about condoms	0.277**	Substance use scale × Knowledge of pregnancy	0.292	Mother’s education: High school or less × Ever had sex	0.0751**
Other race × Attitudes about condoms	−0.342**	In a serious relationship × Refusal skill	0.0710**	Had sex without a condom in past three months × Knowledge of condoms	0.101
Mother’s education: Some college or more × Sexting scale	0.0250	Grade 9 × Mother figure present	−0.0409*	Substance use scale × Knowledge of pregnancy	0.460***
Mother’s education: Some college or more × Ever been in a relationship	0.0215	Negotiating skill × Ever had sex	0.0386	Knowledge of HIV × Ever had sex	0.0547
Age × Kissing scale	−0.00176	Number of partners for vaginal sex × Summer between baseline and follow-up	0.0286***	Knowledge of condoms × Ever had sex	0.115
Age × Ever been in a relationship	0.00327	Knowledge of condoms × Ever had sex	0.0266	Age × Times had sex in past three months	0.000183*
Substance use scale × In a casual relationship	0.387*	Knowledge of HIV × Ever had sex	0.0235		
Substance use scale × Knowledge about condoms	0.213	Substance use scale × Knowledge of condoms	−0.00919		
Substance use scale × Sexting scale	0.0276	Had sex in the past three months × Negotiating skill	0.00500		
In a serious relationship × Attitudes about condoms	0.0651	Knowledge of pregnancy × How many times had sex in last three months	0.00334		
In a serious relationship × Kissing scale	0.0580	Father figure present × How many times had sex in last three months	0.00214		
In a serious relationship × Intent to have vaginal sex in next 12 months	0.0840				
Grade 9 × Summer between baseline and follow-up	−0.0698				
Grade 9 × Girl	−0.0488				
Knowledge about pregnancy × Touching scale	0.0844				
Beliefs about condoms × Touching scale	0.174**				
Attitudes about condoms × Touching scale	0.0629				
Negotiating skill × Sexting scale	0.117				
Negotiating skill × Opportunity scale	0.000446				
Refusal skill × Opportunity scale	0.102				
Sexting scale × Ever been in a relationship	−0.0299				
Touching scale × Intent to have vaginal sex in next 12 months	0.0821				
Opportunity scale × Kissing scale	0.0748				
Grade 11 × In a serious relationship	0.344*				
Kissing scale × Summer between baseline and follow-up	0.0699				
Girl × Mother’s education: High school or less	−0.0709*				
Attracted to same-gender people × Online scale	−0.323**				
**n**	833		1,294		1,282
**R squared**	0.231		0.347		0.286
**Share predicted correctly**	0.801		0.812		0.846

Note: While not shown for brevity and clarity, two cluster variables were selected by lasso for initiation and one for recent sex.

**Table 4:** Proportion of students whose behavior at follow-up we predicted correctly with different models.

	Initiation				Recent sex				Sex without a condom			
	OLS (joint)	Lasso (joint)	Lasso (joint with implicit main effects)	OLS (separate)	OLS (joint)	Lasso (joint)	Lasso (joint with implicit main effects)	OLS (separate)	OLS (joint)	Lasso (joint)	Lasso (joint with implicit main effects)	OLS (separate)
**Total**	0.806	0.801	0.808	0.811	0.812	0.813	0.854	0.846	0.848			
**Boys**	0.773	0.752	0.780	0.798	0.784	0.795	0.799	0.825	0.843	0.812	0.818	0.849
**Girls**	0.820	0.828	0.822	0.835	0.829	0.822	0.821	0.834	0.862	0.870	0.870	0.877
**Black**	0.803	0.795	0.802	0.808	0.806	0.805	0.807	0.818	0.858	0.847	0.848	0.856
**All other races**	0.814	0.820	0.831	0.913	0.827	0.837	0.834	0.876	0.840	0.840	0.847	0.911
**Grade 9**	0.810	0.807	0.822	0.827	0.836	0.834	0.832	0.857	0.900	0.887	0.891	0.907
**Grade 10**	0.801	0.793	0.791	0.801	0.792	0.796	0.798	0.818	0.820	0.817	0.821	0.856
**LGB**	0.833	0.833	0.833	.	0.794	0.794	0.778	.	0.841	0.825	0.810	.
**Non-LGB**	0.804	0.799	0.807	.	0.812	0.813	0.815	.	0.855	0.847	0.850	.

Note: OLS (joint) is the OLS model estimated on all students. Lasso (joint) is the traditional lasso model estimated on all students. Lasso (joint, with implicit main effect) is the lasso model with the addition of all baseline variables that were selected in the interaction terms. OLS (separate) are OLS regressions stratified by the demographic characteristic listed on the left. OLS = Ordinary least squares; LGB = Lesbian, gay, and bisexual.

**Table 5:** Percentage of students by predicted outcome under OLS and lasso models.

Initiation (n = 833)		
	Lasso predicts NO	Lasso predicts YES
OLS predicts No	86.6 %	4.4 %
OLS predicts Yes	2.5 %	6.5 %
**Recent sex (n** = **1,294)**		
	Lasso predicts NO	Lasso predicts YES
OLS predicts No	72.4 %	2.6 %
OLS predicts Yes	2.2 %	22.8 %
**Sex without a condom (n** = **1,282)**		
	Lasso predicts NO	Lasso predicts YES
OLS predicts No	85.4 %	2.3 %
OLS predicts Yes	3.1 %	9.2 %

OLS = Ordinary least squares.

## Data Availability

Data will be made available on request.

## Footnotes

1 We also excluded individuals who gave internally inconsistent responses to key questions across or within survey waves, as described in Cole et al. (2022) available at https://opa.hhs.gov/sites/default/files/2022-06/making-proud-choices-evaluation-may-2022.pdf.

2 The survey asked students to identify their gender. Answer options were “male,” “female” (which are terms used to describe sex assigned at birth, not gender), “transgender” (when gender differs from sex assigned at birth), and “other” with a write-in option. Because the question referred to gender and not sex assigned at birth, we refer to students who identified their gender as “male” as “boys” and students who identified their gender as “female” as “girls.”.

3 Including cluster fixed effects in our specification means that our results will show how variation in covariates for students *within a cluster* predicts variation in outcomes *within a cluster*—in particular, since the MPC treatment was assigned at the cluster level, it will not affect the relationships estimated here so it was not included in the regression. For symmetry, we include cluster fixed effects, but not treatment status, among the candidate covariates for the lasso analysis.
