# Supplementary material for: Predictors of teen sexual behavior
Source: Child Youth Serv Rev. Author manuscript; Available in PMC 2025 Aug 21. (PMC12367029; doi:10.1016/j.childyouth.2023.107247)
Supplement: Supplementary [file NIHMS2050988-supplement-Supplementary.docx]

ONLINE APPENDIX

Table A.1. Measures of pre-sexual and sexual behaviors and other selected outcomes

| **Measure** | **Description** |
| --- | --- |
| Sexting behavior index | Proportion of questions about sending and receiving nude photos to which the respondent answers yes:   - Have you ever sent nude or nearly nude pictures or videos of yourself to others? - Have you ever received nude or nearly nude pictures or videos of someone else? |
| Touching behavior index | Proportion of questions about touching of private parts to which the respondent answers yes:   - Have you ever touched someone’s private parts? Private parts are the parts of the body covered by underwear or a bra. - Other than a doctor or a nurse, have you ever let someone touch your private parts? |
| Opportunity scale | Proportion of questions about opportunities to have sex to which the respondent answers yes:   - Have you ever hung out alone with someone you were attracted to? - Have you ever laid down alone with someone you were attracted to? |
| Kissing behavior scale | Proportion of questions about kissing to which the respondent answers yes:   - Have you ever kissed someone you were attracted to on the mouth? - Have you ever tongue kissed or French kissed someone? |
| Substance use scale | Proportion of substances used in the past 30 days (vape, cigarettes, alcohol, marijuana, other illicit drugs) |
| Ever contacted someone online to meet up in person | Proportion of questions about online contact to which the respondent answers yes:   - In the past three months, have you been contacted by someone you met online to meet in person? - In the past three months, have you contacted someone you met online to try and meet up in person? |

| Ever had sex | | Binary variable representing whether the respondent reported having ever had vaginal, oral, or anal sex   - Have you ever had vaginal sex? - Have you ever had oral sex? - Have you ever had anal sex? - Note, in order to measure sexual initiation among sexually inexperienced youth, we define an additional follow-up variable called “initiation” that is equal to “ever had sex” at follow-up for youth who were not sexually experienced at baseline, and missing otherwise. |
| --- | --- | --- |
| Sexual activity in the last three months | | Binary variable representing whether the respondent reported having vaginal, oral, or anal sex in the three months before completing the follow-up survey   - Now please think about the past three months. In the past three months, have you had vaginal sex, even once? - Now please think about the past three months. In the past three months, have you had oral sex? - Now please think about the past three months. In the past three months, have you had anal sex? |
| Sex without a condom or dental dam in last three months | Binary variable representing whether respondent reported having any risk for STI sex (sex without a condom or dental dam) in the three months before completing the follow-up survey   - In the past three months, how many times have you had vaginal sex without you or your partner using a condom? - In the past three months, how many times have you had oral sex without using a condom or a dental dam? - In the past three months, how many times have you had anal sex without using a condom? | |
| In a relationship | Binary variable indicating if the respondent reporting being in any relationship, including “Engaged,” "In a serious relationship," and "In a casual relationship.” Other relationship options include not currently in a relationship or dating. | |
| Refusal skill | A scale (0-1) measuring a teenager's comfort level in refusing to have sex. | |
| Beliefs about sex | Proportion of statements about how sexual activity will interfere with goals and dreams that the respondent agrees or strongly agrees with:  • Having sex at your age would make you less likely to have the career you are hoping for.  • Having sex at your age would make you less likely to graduate from high school. | |
